# Increased urinary Smad3 is significantly correlated with glomerular hyperfiltration and a reduced glomerular filtration rate and is a new urinary biomarker for diabetic nephropathy

**DOI:** 10.1186/s12882-015-0156-8

**Published:** 2015-10-08

**Authors:** Kaifeng Guo, Junxi Lu, Jingxin Kou, Mian Wu, Lei Zhang, Haoyong Yu, Mingliang Zhang, Yuqian Bao, Haibing Chen, Weiping Jia

**Affiliations:** Department of Endocrinology and Metabolism, Shanghai Clinical Center for Diabetes, Shanghai Diabetes Institute, Shanghai Key Laboratory of Diabetes Mellitus, Shanghai Jiaotong University Affiliated Sixth People’s Hospital, Shanghai, 200233 China; Department of Rheumatology, Taixing people’s Hospital, Taixing, Jiangsu 225400 China

**Keywords:** Smad3, Glomerular hyperfiltration, Diabetic nephropathy, Type 2 diabetes

## Abstract

**Background:**

Diabetic nephropathy is one of the major microvascular complications of diabetes. We investigated the association between urinary Smad3 (usmad3) levels, glomerular hyperfiltration, and the development of nephropathy in patients with type 2 diabetes mellitus (T2DM).

**Methods:**

The usmad3 level was determined by enzyme-linked immunosorbent assay in 245 well-characterised patients with T2DM and 82 healthy control subjects. The associations of the usmad3 level with glomerular hyperfiltration, glucose and lipid profiles, and renal function were evaluated.

**Results:**

The usmad3 level was significantly higher in patients with diabetes than in the control group. The level in the hyperfiltration group was higher than that in the normofiltering group, regardless of whether patients were in the normoalbuminuric or the proteinuria groups. Pearson’s correlation analysis suggested that the usmad3 level was significantly correlated with age, systolic blood pressure, fasting plasma glucose, insulin, C-peptide, glycated haemoglobin, and estimated glomerular filtration rate (eGFR). A multiple linear stepwise regression analysis revealed that usmad3 levels in patients with T2DM and an eGFR ≥90 ml/min/1.73 m^2^ were independently and positively correlated with eGFR, whereas in patients with T2DM and eGFR <90 ml/min/1.73 m^2^, the levels were independently and negatively correlated with eGFR.

**Conclusions:**

The usmad3 level was significantly correlated with biphasic changes in the GFR (both glomerular hyperfiltration and reduced eGFR) in patients with T2DM. Usmad3 may serve as a novel marker for hyperfiltration and for screening patients with T2DM for nephropathy.

## Background

Diabetic nephropathy (DN) is the leading cause of end-stage renal disease in developed and developing countries. Therefore, identifying the earliest markers and risk factors linked to progression of this disease is important. Currently, albuminuria is the most widely used and accepted marker of DN in clinical practice. Unfortunately, significant glomerular lesions can be present in normoalbuminuric patients [1]. In addition, structural renal lesions are often already present when microalbuminuria is detected [2, 3]. Therefore, the quest for more reliable renal biomarkers with higher sensitivity and specificity are needed to predict early onset DN and monitor its progression. Furthermore, such biomarkers could provide better insight into identifying the complex pathophysiological processes responsible for DN.

Early changes in DN include increases in kidney size, glomerular volume, and glomerular filtration rate (GFR), followed by the accumulation of glomerular extracellular matrix, increased urinary albumin excretion, glomerular sclerosis and tubular fibrosis. Late-stage overt DN is clinically characterised by proteinuria, hypertension and progressive renal insufficiency [4–6]. In humans, glomerular hyperfiltration associated with early DN is a risk factor for development of progressive DN [7]. Hyperfiltration is typically defined by an estimated glomerular filtration rate (eGFR) of 125–140 mL/min/1.73 m^2^, or greater than two standard deviations above the mean eGFR in normal, healthy individuals [8, 9]. The potential mechanisms leading to the development of glomerular hyperfiltration in patients with type 2 diabetes mellitus (T2DM) are not fully understood, but they may involve glomerular haemodynamic and tubular factors [10]. These haemodynamic changes are associated with activation of pro-inflammatory cytokines, such as transforming growth factor-β (TGF-β), leading to proteinuria and kidney disease [11, 12].

TGF-β/Smad signalling is a key pathway in the pathogenesis of DN [13] and is highly activated in DN patients, as identified by nuclear translocation of phosphorylated Smad2 and Smad3 in glomerular and tubulointerstitial cells. Activation of this pathway significantly increases Smad3 and Smad2 expression in the kidneys and contributes significantly to both glomerular and interstitial fibrosis [14]. However, recent studies have found that Smad3, but not Smad2, mediates renal fibrosis under different disease conditions, including diabetes, and may play a more important role in the pathogenesis of DN [15]. Albuminuria and the eGFR are both important indicators in the diagnosis of DN. Our previous study found that the urinary Smad3 (usmad3) level is significantly correlated with proteinuria in patients with DN [16]. However, whether usmad3 is associated with the GFR (hyperfiltration or eGFR reduction or both) remains unknown. Smad3 plays an important role in the morphological changes that occur during DN and may be related to glomerular hyperfiltration. One study found that usmad1 (bone morphogenetic protein-specific R-Smads) increases significantly in patients with glomerular hyperfiltration and T2DM [17], suggesting that smad3 may have the same effect. Therefore, we further investigated whether usmad3 is associated with the eGFR in patients with T2DM and analysed the clinical factors related to usmad3.

## Methods

### Study population

Subjects with T2DM (*n* = 245) and their respective age- and sex-matched healthy volunteers (*n* = 82) were recruited from the out-patient clinic at the Shanghai Clinical Centre for Diabetes (Shanghai, China). The diagnosis of diabetes mellitus was performed according to the World Health Organization (WHO) study group (1999) criteria. Patients and controls were interviewed personally by the investigators to exclude acute complications of diabetes mellitus, a history of non-diabetic renal disease, urinary tract infection, symptoms or history of heart disease, and acute or severe chronic liver disease. All patients with T2DM were treated with oral antidiabetic drugs. The study was conducted in accordance with the Declaration of Helsinki and approved by the Ethics Committee of Shanghai Jiao Tong University Affiliated Sixth People’s Hospital. All study participants provided written informed consent prior to enrolment.

### Anthropometric and biochemical measurements

Demographic and clinical data, including age, sex, diabetes duration, weight and height, were recorded. Blood pressure was measured twice using a Hawksley sphygmomanometer after 10 min of supine rest, and the average blood pressure was calculated using the formula: (systolic blood pressure [SBP] + 2 (diastolic blood pressure)/3. Venous blood samples were collected between 08:00 and 09:00 h after a 12-h fast. Serum samples were separated by centrifugation at 4 °C at 4000 g for 10 min. Fasting blood samples were collected to determine fasting plasma glucose (FPG), glycosylated haemoglobin (HbA1c) and serum lipid, lipoprotein and creatinine levels. Body mass index (BMI) was calculated as weight divided by height squared (kg/m^2^). Plasma glucose levels were measured in the fasting state (i.e., FPG) and in the 2-h postprandial state (2hPG) using the glucose oxidase method. HbA1c was determined using the Bio-Rad Variant II analyser (Bio-Rad Laboratories Inc., Hercules, CA, USA). Serum urea nitrogen, creatinine, uric acid, and lipid profiles, including measurements of total cholesterol (TC), triglycerides, high density lipoprotein and low density lipoprotein (LDL) were measured on a Hitachi 7600 analyser using an enzymatic assay (Hitachi Inc., Tokyo, Japan). Serum high-sensitive C-reactive protein was measured by a particle-enhanced immunoturbidimetric assay (Dade Behring Inc., Newark, NJ, USA). Urinary albumin was determined using a Dade Behring BN II analyser by nephelometry (N antiserum in the Human Albumin Assay, Dade Behring, Glasgow, DE, USA). Serum creatinine and urinary creatinine concentration were measured on a Hitachi 7600 analyser using the sarcosine oxidase-PAP method. The urinary albumin:creatinine ratio (UACR) was computed and reported in mg/g (1 mg/g = 0.131 mg/mmol). The albuminuria categories included normoalbuminuria, microalbuminuria and macroalbuminuria defined as an ACR <30, 30–299, and ≥300 mg/g, respectively. The eGFR was estimated using the Modification of Diet in Renal Disease equation: 186 × [serum creatinine (mg/dL)]^−1.154^ × (age)^−0.203^ × (0.742 if female). The eGFR categories included ≥131, 90–130, 60–89, and <60 ml/min/1.73 m^2^.

### Hyperfiltration definition

Hyperfiltration has largely been considered a dichotomous variable in the academic literature, with a threshold of 125–140 ml/min/1.73 m^2^, depending on the population evaluated and the formula used to estimate kidney function. We defined hyperfiltration as an eGFR ≥131 ml/min/1.73 m^2^ (mean ± 2 standard deviations) in accordance with age- and sex-specific healthy subjects, and an eGFR of 90–130 ml/min/1.73 m^2^ was defined as normofiltering.

### Determination of urinary Smad3 concentration

Freshly voided urine was collected from patients during their routine visits to Shanghai Clinical Centre for Diabetes. During the same period, samples were obtained from healthy volunteers with no history of diabetes, hypertension, or renal disease. Urine samples were maintained at −80 °C and were later thawed, centrifuged, and assayed directly by enzyme-linked immunoassay (ELISA). The usmad3 measurements were performed using a commercially available ELISA kit (USCN Life Science and Technology Co., Wuhan, Hubei, PRC) according to the manufacturer’s protocol. The detection limit in the human usmad3 assay was 0.159 ng/ml, and the intra- and interassay coefficients of variations were 6.7 % and 8.5 %, respectively. Duplicate measurements were obtained for all samples. The usmad3 level was expressed as a ratio relative to the creatinine level (ng/mmol creatinine). Serial dilutions of recombinant smad3 were included in all assays as standards.

#### Statistical analyses

All statistical analyses were performed by SPSS ver. 17.0 software (IBM Inc., Chicago, IL, USA). Data were presented by descriptive analysis [mean ± SD for normal distribution or medians (lower quartile – upper quartile) for non-normal distribution]. Data that were not normally distributed, such as the smad3 level, as determined by the Kolmogorov–Smirnov test, were logarithmically transformed before analysis. However, mean values of the variables are presented as untransformed data to aid interpretation. Student’s unpaired *t*-tests were used to compare two groups. Analysis of variance with post-hoc analysis and Bonferroni correction were used to identify differences between groups. Pearson’s correlation analysis was used when appropriate for comparisons between groups, and multiple testing was adjusted using Bonferroni correction. Multivariate stepwise linear regression analysis was conducted using the dependent variable, smad3, and those variables showing a significant correlation with smad3 as the independent variables. A *p-*value <0.05 was considered significant.

## Results

### Subject characteristics

Anthropometric and metabolic characteristics of all participants in the cross-sectional study are summarized in Table 1. There were no statistically significant sex differences between the groups of control (male, 52.4 %) and T2DM (male, 58.0 %), *p* = 0.383. Moreover, the mean age did not differ significantly between the control (57.1 ± 9.4 years) and T2DM subjects (58.1 ± 9.9 years), *p* = 0.461. Patients with T2DM had higher levels of HbA1c, FBG and 2hPG compared with the controls. Hyperfiltration (16.3 %) occurred more often in younger patients with T2DM who had a shorter diabetes duration and higher creatinine, uric acid and HbA1c levels, compared with the normofiltering group. However, BMI, blood pressure and blood lipids were similar between those with and without hyperfiltration.

**Table 1:** Clinical characteristics of healthy individuals and diabetic patients according to level of eGFR

	Controls	T2DM patients			
		90–130	≥131	60–89	<60
N	82	84	40	79	42
Age (years)	57.1 ± 9.3	55.5 ± 7.4	54.9 ± 9.0	60.6 ± 11.0*	61.6 ± 11.2*
Male/Female	43/39	46/38	20/20	50/29	26/16
BMI(Kg/m^2^)	23.89 ± 2.54	25.90 ± 3.38*	25.97 ± 3.93*	26.11 ± 3.56*	25.45 ± 4.40*
Diabetes duration(years)	—	8.01 ± 6.45	6.47 ± 5.35^#^	9.95 ± 7.65^#^	11.67 ± 8.23^#^
HbA1c ,%	5.3 ± 0.24	9.06 ± 1.92*	9.92 ± 1.83*^#^	9.37 ± 2.55*	9.10 ± 2.69*
FPG(mmol/L)	5.04 ± 0.36	8.84 ± 3.00*	9.60 ± 2.96*	9.86 ± 2.91*^#^	9.41 ± 3.00*
2hPG(mmol/L)	6.06 ± 1.05	14.63 ± 5.13*	15.98 ± 5.11*	13.45 ± 4.17*	13.90 ± 4.57*
Insulin(mU/l)	9.73 ± 7.11	25.26 ± 23.11*	19.03 ± 26.02	23.47 ± 30.50*	34.69 ± 20.90*
C-peptide(μg/l)	1.79 ± 1.01	2.09 ± 1.03	1.69 ± 0.99	2.38 ± 1.52*	2.92 ± 1.43*^#^
SBP(mmHg)	126.8 ± 16.1	134.2 ± 14.4*	132.0 ± 14.0	137.6 ± 15.8*	148.1 ± 23.8*^#^
DBP(mmHg)	79.8 ± 10.5	83.0 ± 9.2	80.6 ± 8.7	80.3 ± 10.7	83.3 ± 13.7
TC(mmol/L)	5.05 ± 0.74	4.72 ± 1.10	4.82 ± 1.00	5.02 ± 1.50	5.05 ± 1.55
TG(mmol/L)	1.53 ± 0.99	2.13 ± 2.15*	1.85 ± 1.39	2.23 ± 2.12*	2.26 ± 1.69*
LDL-c (mmol/L)	3.16 ± 0.83	3.14 ± 0.91	3.25 ± 0.97	3.23 ± 1.04	3.21 ± 1.34
HDL-c(mmol/L)	1.45 ± 0.36	1.14 ± 0.45*	1.23 ± 0.27*	1.16 ± 0.32*	1.18 ± 0.49*
hsCRP (mg/L)	1.23 ± 1.20	1.44 ± 1.23	1.66 ± 1.40	2.31 ± 2.40*^#^	2.95 ± 2.15*^#^
Bun (mmol/L)	4.60 ± 1.14	5.61 ± 1.33*	5.16 ± 1.39	6.52 ± 1.84*^#^	10.10 ± 4.90*^#^
Creatinine (μmol/L)	71.94 ± 14.19	62.99 ± 9.85*	47.63 ± 7.97*^#^	86.72 ± 12.90*^#^	140.76 ± 53.33*^#^
Uric acid(μmol/L)	332.7 ± 97.1	321.9 ± 69.0	280.7 ± 77.0*^#^	360.8 ± 92.4^#^	399.5 ± 120.6*^#^
UACR(mg/g)	6.11(4.18–9.51)	21.63(7.57–88.09) *	18.71(8.76–65.79)	86.01(17.74–245.34) *^#^	331.03(188.77–1447.82) *^#^
Microalbuminuria, n (%)	—	33(39.3)	14(35.0)	36(45.6)	18(42.9)
Albuminuria	—	40(47.6)	16(40)	54(68.4)	40(95.2)
eGFR(ml/min/1.73 m2)	94.9 ± 20.1	108.2 ± 11.7*	150.9 ± 21.4*^#^	74.7 ± 8.7*^#^	44.9 ± 12.7*^#^
Lipid-lowering therapy, ( %)	3.7	14.3*	12.5*	22.8*^#^	26.2*^#^
Anti-hypertensives, ( %)	6.1	17.9*	20.0*	25.3*^#^	35.7*^#^
Usmad3 (ng/mmol creatinine)	286.1(177.9–474.8)	314.7(223.4–568.0)	524.5(320.0–1078.1) *^#^	527.9(272.9–1327.5) *^#^	656.6(391.4–2976.0) *^#^

*vs.norm-GFR* group(90–130), *SBP* systolic blood pressure, *DBP* diastolic blood pressure, *BMI* Body mass index, *HbA1c* hemoglobin A1c, *FPG* fasting plasma glucose, *2hPG* 2-h plasma glucose concentration, *TC*, total cholesterol, *TG* triglyceride, *HDL-c*, high density lipoprotein cholesterol; *LDL-c* low density lipoprotein cholesterol

Data are expressed as means ± SD or median (IQR);*P <0.05 vs. controls;^#^ *P* <0.05

### Urinary smad3 in relation to eGFR and albuminuria

**Fig. 1:**
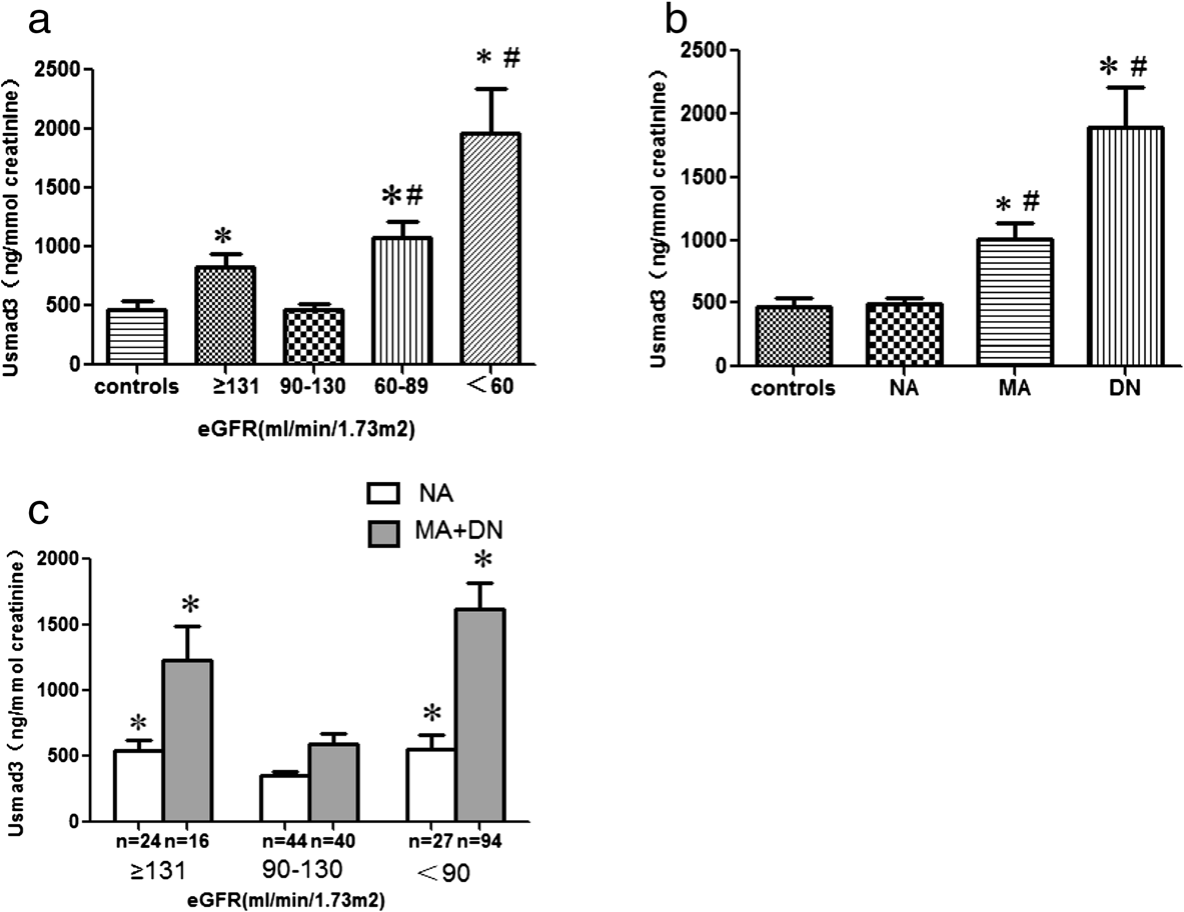
Urinary smad3 in control subjects and patients with type 2 diabetes mellitus (T2DM). **a** Patients with T2DM were divided into four subgroups according to the estimated glomerular filtration rate (eGFR) with values of ≥131, 90–130, 60–89 and <60 ml/min/1.73 m^2^. **b** Patients with T2DM were divided into three subgroups according to albuminuria, including normoalbuminuria, microalbuminuria and macroalbuminuria. **c** Urinary smad3 levels in 95 normoalbuminuria and 150 microalbuminuria and macroalbuminuria patients with T2DM according to the eGFR. *P <0.05 vs. controls; ^#^ *P* <0.05 vs. normal eGFR group (90–130 ml/min/1.73 m^2^)

Usmad3 levels were significantly higher in patients with diabetes than in the control group (976.04 ± 89.16 vs. 469.55 ± 68.99 ng/mmol creatinine, *p* <0.05). No difference in the usmad3 level was observed between males (848.82 ± 102.08 ng/mmol creatinine, *n* = 181) and females (849.30 ± 92.99 ng/mmol creatinine, *n* = 146; *p* = 0.901). The usmad3 level was higher in the hyperfiltration group than in the normofiltering group and healthy control subjects (Table 1, Fig. 1a). Moreover, the usmad3 level was also higher when the eGFR was <90 ml/min/1.73 m^2^ than that in the normofiltering group and healthy control subjects (Table 1, Fig. 1a). In addition, the usmad3 level was significantly higher in the MA group than in the NA group (1004.81 ± 125.08 vs. 491.92 ± 45.26 ng/mmol creatinine; *p* <0.05), whereas no difference was observed in the usmad3 level between the control and NA groups (470.23 ± 70.72 vs. 491.92 ± 45.26 ng/mmol creatinine, *p* <0.05) (Fig. 1b).

Moreover, we performed a subgroup analysis based on the presence or absence of microalbuminuria to further demonstrate the relationship between usmad3 and eGFR, because proteinuria is an important cause of the increase in usmad3. Twenty-four (25.3 %) of the 95 normoalbuminuric patients had a corresponding eGFR ≥131 ml/min/1.73 m^2^, 44 (46.3 %) had an eGFR of 90–130 ml/min/1.73 m^2^, and 27 (28.4 %) had an eGFR <90 ml/min/1.73 m^2^. The usmad3 levels in subjects with normoalbuminuria and hyperfiltration (546.24 ± 71.83 ng/mmol creatinine) and in subjects with an eGFR <90 ml/min/1.73 m^2^ (553.24 ± 112.84 ng/mmol creatinine) were both significantly higher than the levels in subjects with normofiltering (351.92 ± 30.88 ng/mmol creatinine, *p* <0.05) (Fig. 1c). Similarly, usmad3 levels in the 150 patients with microalbuminuria and hyperfiltration (1232.83 ± 257.63 ng/mmol creatinine) and in subjects with an eGFR <90 ml/min/1.73 m^2^ (1621.00 ± 203.32 ng/mmol creatinine) were also both significantly higher than the levels in subjects with normofiltering (587.44 ± 86.50 ng/mmol creatinine; *p* <0.05) (Fig. 1c).

### Associations among urinary smad3 levels, anthropometric parameters, and biochemical indices

**Fig. 2:**
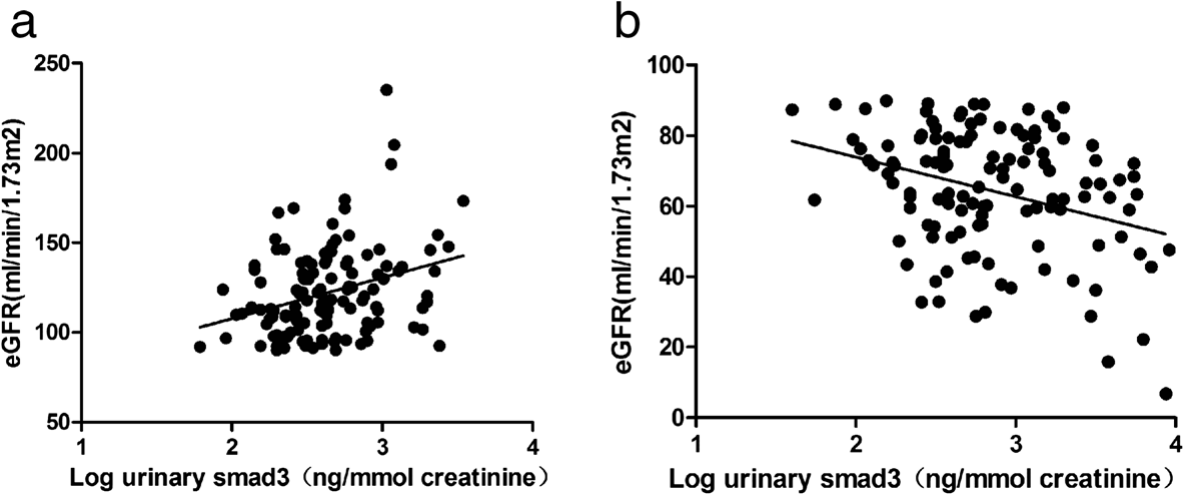
Correlation between urinary smad3 levels (log transformed) in patients with type 2 diabetes mellitus with (**a**) an estimated glomerular filtration rate (eGFR) ≥90 ml/min/1.73 m^2^ or (**b**) an eGFR <90 ml/min/1.73 m^2^

We investigated the relationships of usmad3 levels with a cluster of anthropometric parameters and biochemical indices (Table 2). Pearson’s correlation analysis suggested that the usmad3 level was positively correlated with age (*r* = 0.218, *p* <0.01), SBP (*r* = 0.164, *p* <0.01), FPG (*r* = 0.119, *p* <0.05), 2hPG (*r* = 0.124, p <0.05), insulin (*r* = 0.205, <0.01), C-peptide (*r* = 0.163, *p* <0.05), HbA1c (*r* = 0.153, *p* <0.01), UACR (*r* = 0.553, *p* <0.01), TC (*r* = 0.193, *p* <0.01) and LDL (*r* = 0.195, *p* <0.01), but negatively correlated with eGFR (*r* = −0.267, *p* <0.01) in all 327 subjects. Furthermore, usmad3 levels were associated with HbA1c, eGFR, UACR, TC and LDL after adjusting for age (Table 2). The usmad3 level increased significantly regardless of whether the eGFR was higher or lower than normal. We performed a correlation analysis between usmad3 and different eGFR levels. We found that usmad3 levels in patients with T2DM and an eGFR ≥90 ml/min/1.73 m^2^ were positively correlated with the eGFR (*r* = 0.313, *p* <0.001) (Fig. 2a), whereas they were negatively correlated with the eGFR (*r* = −0.370, *p* <0.001) in patients with T2DM and an eGFR <90 ml/min/1.73 m^2^ (Fig. 2b).

**Table 2:** Correlations of urinary smad3 levels with other variables, unadjusted and after adjusting for age in total 327 subjects

Variables	Urinary smad3			
	Pearson correlation		Pearson correlation adjusted for age	
	r	p	r	p
Age	0.218	<0.001		
SBP	0.164	0.003	0.042	0.530
FPG	0.119	0.040	0.037	0.575
2hPG	0.124	0.037	0.109	0.100
Insulin	0.205	0.001	0.088	1.182
C-peptide	0.163	0.010	0.083	0.212
HbA1c	0.153	0.006	0.167	0.011
eGFR	−0.267	<0.001	−0.252	<0.001
Bun	0.360	<0.001	0.184	0.005
Creatinine	0.309	<0.001	0.187	0.005
UACR	0.553	<0.001	0.473	<0.001
TC	0.193	0.001	0.214	0.001
LDL-c	0.195	0.001	0.134	0.043

*SBP* systolic blood pressure, *HbA1c* hemoglobin A1c, *FPG* fasting plasma glucose, *2hPG*, 2-h plasma glucose concentration; TC, total cholesterol; LDL-c, low density lipoprotein cholesterol

We selected usmad3 as a dependent variable and other clinical parameters as independent variables to build a multiple linear stepwise regression equation and determine the independent relationships between usmad3 and the clinical parameters. Only the variables significantly related to usmad3 in Pearson’s correlation analyses were entered into the multiple linear stepwise regression analysis. The multiple linear stepwise regression analysis was performed in two stages based on the eGFR, because the relationship between usmad3 and eGFR depends on the eGFR. The results revealed that usmad3 levels were independently correlated with eGFR (standardised β = 0.373; *t* = 3.527; *p* = 0.001) in patients with T2DM and an eGFR ≥90 ml/min/1.73 m^2^, whereas they were also independently correlated with eGFR in patients with T2DM and an eGFR <90 ml/min/1.73 m^2^ (standardised β = −0.305; t = −2.696; *p* = 0.009).

## Discussion

Cytokines, particularly TGF-β and monocyte chemotactic protein 1, have been studied extensively in patients with DN [18]. Serum cytokines reflect systemic inflammation, whereas urinary cytokines may be biomarkers of renal damage. In the current study, we evaluated usmad3 concentrations in normal subjects and patients with T2DM and investigated the associations of usmad3 levels with glomerular hyperfiltration and albuminuria, two important features of DN. The novelty of our study is that we demonstrated for the first time that the usmad3 level was significantly correlated with biphasic changes in GFR (both glomerular hyperfiltration and eGFR reduction). This result and our previous study illustrate that usmad3 may be an important DN marker.

TGF-β is a key mediator in the development of diabetic complications. Smad2 and Smad3 are TGF-β/activin-specific R-Smads, which are strongly activated in both experimental and human DN. However, recent studies have found that Smad3 plays a more critical role in the pathogenesis of DN than does Smad2. Smad3, but not Smad2, may directly mediate transcription of genes associated with the collagen matrix [19], epithelial-myofibroblast transition [20], endothelial-myofibroblast transition [21], connective tissue growth factor [22] and vascular endothelial growth factor [23] during development of DN. Deleting Smad3, but not Smad2, inhibits these effects. Thus, these findings suggest that the Smad3-mediated signalling pathway may be a key mechanism leading to DN.

Glomerular hyperfiltration associated with early DM in humans is a risk factor for development of progressive DN, which is seen in 25–75 % of patients with type 1 diabetes mellitus (T1DM) and may be affected by factors such as age, diabetes duration and glycaemic control [9]. The occurrence of hyperfiltration in patients with T2DM (range 5–40 %) is likely to be lower than in T1DM [9, 24]. The pathogenesis of hyperfiltration remains controversial, as both haemodynamic (‘haemodynamic hypothesis’) and tubuloglomerular feedback mechanisms (‘tubular hypothesis’) have been implicated [10]. Given the deleterious effects of renal hyperfiltration on the risk of DN and the possible clinical benefit derived by reducing intraglomerular pressure, it is of utmost clinical importance to discover DN early [25].

Our results demonstrate for the first time that usmad3 is highly expressed in patients with T2DM and hyperfiltration. The hyperfiltration mechanism that promotes usmad3 expression is incompletely understood, but may be related to renal upregulation of TGF-β. Renal hyperfiltration due to haemodynamic function abnormalities is seen in animal models of T2DM, including increased intraglomerular capillary pressure and glomerular hyperfiltration [11, 26]. Activation of pro-inflammatory cytokines, such as TGF-β, leading to proteinuria and kidney disease is associated with these haemodynamic changes [12, 27]. The high TGF-β expression induced by haemodynamic abnormalities during hyperfiltration may increase production of downstream molecular pathways and further production of Smad3. The signalling of TGF-β/Smad is highly activated in patients with DN and contributes significantly to both glomerular and interstitial fibrosis. These symptoms have also been seen in an experimental animal model of T1DM kidney disease [28]. Thus, the Smad signalling pathway is dysregulated and imbalanced in the diabetic kidney.

Expression of usmad3 was also increased when the eGFR fell below 90 ml/min/1.73 m^2^, and usmad3 was higher than that in the normofiltering group and healthy control subjects. A greater eGFR decline is associated with more advanced diabetic glomerulopathy and worse metabolic control [29]. In addition, the severity of proteinuria was significantly associated with the usmad3 level in our study. Levels of usmad3 were significantly higher in the macro- and microalbuminuria groups than in the normoalbuminuric group. Moreover, we also found that the usmad3 level in subjects with hyperfiltration was also significantly higher than that in normofiltering and normoalbuminuric patients with T2DM. Previous work in patients with T1DM has demonstrated that urinary inflammatory mediators correlate with reduced eGFR and with increased urinary albumin excretion, even when ACR values are normal [30]. Since hyperfiltration is associated with DN and occurs early in the natural history of diabetes before the onset of microalbuminuria, our results may identify a potentially high-risk group that could benefit from earlier renoprotective therapies.

Pearson’s correlation analysis demonstrated significant correlations between usmad3 and SBP, HbA1c, eGFR, UACR, TC, and LDL. In particular, we performed a correlation analysis between usmad3 and the eGFR according to different eGFR levels. Levels of usmad3 in patients with T2DM and an eGFR ≥90 ml/min/1.73 m^2^ were positively correlated with eGFR, whereas patients with T2DM and an eGFR of <90 ml/min/1.73 m^2^ were negatively correlated. Furthermore, a multiple linear stepwise regression analysis revealed that usmad3 levels in patients with T2DM and an eGFR of ≥90 ml/min/1.73 m^2^ were independently correlated with the eGFR, whereas usmad3 was also independently correlated with the eGFR in patients with T2DM and an eGFR of <90 ml/min/1.73 m^2^. These results show that the smad3 level was significantly correlated with eGFR at two important stages during DN progression (hyperfiltration and GFR decline) and may reflect the progress of DN.

Our study had several limitations. First, the small sample size may have limited our ability to detect between-group differences in usmad3 excretion. The number of patients with glomerular hyperfiltration was limited, which may have weakened the association between usmad3 and glomerular hyperfiltration. Second, our study design was cross-sectional and did not address the cause-effect relationship between usmad3 and DN. Third, we used MDRD to estimate GFR rather than measuring GFR using iothalamate clearance to determine kidney function, which may show a poorer performance compared to more accurate methods. Finally, although it is presumed that hyperfiltration may have induced the increase in cytokines and ultimately to renal injury, it is possible that other factors affecting urinary cytokine excretion could have induced hyperfiltration. Therefore, it is important for future studies to clarify the time course and reversibility of urinary biomarkers using existing and investigational agents. Future work should determine whether usmad3 excretion in the hyperfiltration group is systemically-derived (i.e., ‘spill-over’) or due to renal production.

## Conclusions

In summary, this is the first clinical study to demonstrate that usmad3 is significantly correlated with biphasic changes in the GFR (both glomerular hyperfiltration and eGFR reduction) in patients with T2DM. Usmad3 may serve as an early marker of glomerular hyperfiltration, useful in the screening of nephropathy among patients with T2DM. A large, prospective, multicentre trial is needed to assess the performance of this biomarker and should enlist patients with type 1 and T2DM with and without DN to identify its role in clinical practice.

## Abbreviations

DN: Diabetic nephropathy
GFR: Glomerular filtration rate
MDRD: Modification of diet in renal disease
T2DM: Type 2 Diabetes Mellitus
